# Dr. Harry A. Powers

**Published:** 1898-12

**Authors:** 


### OBITUARY.

Dr. Harry A. Powers, of Buffalo, died at the Fitch Hospital, October 24, 1898, aged 28 years. The circumstances attending the tragic death of this bright and promising young physician are indeed painful. It appears that of late he manifested a disposition to brood over trifles and became despondent without apparent cause. He was prospering in his profession to an unusual degree for a young physician. However, late Saturday evening, October 22d, he took twelve grains of morphine, as has since been ascertained, or as he himself announced at the time. Immediately medical aid was summoned, but meanwhile, desperately determining to end his life, Dr. Powers had shot himself three times with a revolver, the pistol and three empty shells testifying to the sad deed. Dr. Grosvenor R. Trowbridge, who came to his succor, found the wounds and evidences of morphinism present. Next morning Dr. Trowbridge had his patient removed to the Fitch Hospital, where Dr. Powers had formerly performed service as house surgeon. He lingered until 7

o'clock p. m. Monday—nearly forty-eight hours after taking the poison and inflicting the wounds—when he passed away.

Dr. Powers was a native of St. Johnsbury, Vt., graduated at Dartmouth College, and then came to Buffalo to obtain his medical education, receiving his doctorate degree from the University of Buffalo in 1895. He located here for practice, and his father and mother, who were members of his household, deserve and receive a widespread sympathy in their affliction.

